# Urine VCAM-1 as a marker of renal pathology activity index in lupus nephritis

**DOI:** 10.1186/ar3912

**Published:** 2012-07-13

**Authors:** Sandeep Singh, Tianfu Wu, Chun Xie, Kamala Vanarsa, Jie Han, Tina Mahajan, Ho Bing Oei, Chul Ahn, Xin J Zhou, Chaim Putterman, Ramesh Saxena, Chandra Mohan

**Affiliations:** 1 Department of Internal Medicine/Rheumatology, University of Texas Southwestern Medical Center, 5323 Harry Hines Boulevard, Dallas, TX 75390, USA; 2 Department of Internal Medicine/Rheumatology, Albert Einstein College of Medicine, 1300 Morris Park Avenue, Bronx, NY 10461, USA

## Abstract

**Introduction:**

Although renal pathology is highly predictive of the disease course in lupus nephritis, it cannot be performed serially because of its invasive nature and associated morbidity. The goal of this study is to investigate whether urinary levels of CXC ligand 16 (CXCL16), monocyte chemotactic protein-1 (MCP-1) or vascular cell adhesion molecule-1 (VCAM-1) in patients with lupus nephritis are predictive of particular features of renal pathology in renal biopsies obtained on the day of urine procurement.

**Methods:**

CXCL16, MCP-1, and VCAM-1 levels were measured in urine samples from 74 lupus nephritis patients and 13 healthy volunteers. Of the patients enrolled, 24 patients had a concomitant kidney biopsy performed at the time of urine collection. In addition, patients with other renal diatheses were also included as controls.

**Results:**

All three molecules were elevated in the urine of systemic lupus erythematosus patients, although VCAM-1 (area under curve = 0.92) and MCP-1 (area under curve = 0.87) were best at distinguishing the systemic lupus erythematosus samples from the healthy controls, and were also most strongly associated with clinical disease severity and active renal disease. For patients in whom concurrent renal biopsies had also been performed, urine VCAM-1 exhibited the strongest association with the renal pathology activity index and glomerulonephritis class IV, although it correlated negatively with the chronicity index. Interestingly, urinary VCAM-1 was also elevated in anti-neutrophil cytoplasmic antibodies-associated glomerulonephritis, focal segmental glomerulosclerosis and membranous nephropathy but not in minimal-change disease.

**Conclusion:**

Urinary VCAM-1 emerges as a reliable indicator of the activity:chronicity ratios that mark the underlying renal pathology in lupus nephritis. Since VCAM-1 is involved in the acute phase of inflammation when leukocytic infiltration is ongoing, longitudinal studies are warranted to establish whether tracking urine VCAM-1 levels may help monitor clinical and pathological disease activity over time.

## Introduction

Systemic lupus erythematosus (SLE) is a prototypic autoimmune disease with the potential to affect a variety of end organs. Lupus nephritis (LN) is one of the most frequent manifestations of SLE and can be present in 60% of SLE patients [1]. LN is associated with significant morbidity and mortality and is the most common secondary glomerulonephritis leading to end-stage renal disease [2]. Patients with end-stage renal disease require supportive therapy with dialysis or need to undergo renal transplantation, amounting to a huge burden on our healthcare system. Early diagnosis and prompt treatment of LN is associated with significantly better outcome [3]. Kidney biopsy remains the mainstay of LN diagnosis and is usually prompted by an abnormal urinary sediment, proteinuria or elevated serum creatinine. These markers are crude and do not always correlate with histopathological diagnosis. The same markers are used to assess treatment response as well as to detect nephritic flares but they are not very accurate and do not match the predictive value of a kidney biopsy. Renal damage is known to precede the appearance of proteinuria, elevation of serum creatinine or abnormal urine sediment. This leads to a delay in diagnosis of and instituting treatment of LN or its flares and the assessment of treatment response, thus contributing to significant morbidity and mortality [4,5]. There is hence an urgent need for the identification of surrogate biomarkers that closely parallel renal pathology in lupus and will prompt us to perform a renal biopsy early in the course of disease so that induction therapy can be instituted promptly.

Several studies of murine models of LN as well as studies in SLE patients have uncovered a number of potential disease biomarkers - including chemokines, cytokines and adhesion molecules - that may correlate well with LN [6-9]. However, few of these studies have attempted to correlate the urinary biomarker levels with concurrent renal pathology [6-12]. This study was designed to address this knowledge gap, with a focus on three promising biomarkers.

Monocyte chemotactic protein-1 (MCP-1), vascular cell adhesion molecule-1 (VCAM-1) and CXC ligand 16 (CXCL16) have been documented to be increased within the kidneys, sera and urine of SLE patients and seem to correlate with disease activity, although their clinical utility in predicting disease activity in LN remains to be fully established [6,7,9,12-15]. MCP-1 is a chemotactic factor involved in leukocyte recruitment to the kidney. In a murine model of LN, MRL.lpr mice engineered to lack MCP-1 or subjected to pharmacological blockade of MCP-1 exhibited prolonged survival with reduced renal damage [16]. Moreover, urinary MCP-1 levels have been shown to be elevated in patients with active LN at the time of renal flares, and these levels tend to recede with successful treatment of LN [6,12,14]. Furthermore, increased glomerular expression of MCP-1 appears to be predictive of poor renal prognosis in pediatric LN [17].

VCAM-1 is an adhesion molecule involved in the migration and recruitment of inflammatory cells through its interaction with an integrin (very late antigen-4) present on infiltrating leukocytes. In murine lupus, VCAM-1 has been shown to be hyperexpressed in the endothelium, in the glomeruli and in the tubules of MRL.lpr mice [18], as well as on myeloid cells in mice bearing particular lupus susceptibility loci [19]. The production of VCAM-1 as well as serum levels of this molecule are increased in patients with SLE [20], and this is even more pronounced in LN, particularly in proliferative class III and class IV LN [10,11]. Furthermore, the urinary levels of VCAM-1 are also increased in SLE and LN patients, and tend to correlate with various activity parameters [13,21]. Finally, the documented urinary enrichment of VCAM-1 relative to the serum levels suggests it may be partly renal in origin [9]. Similarly, we have previously documented that the serum and urine levels of the chemokine CXCL16 are also increased in human SLE as well as in murine lupus, with increased expression being observed within the kidneys [9]. An independent report has noted the increased gene upregulation of CXCL16 in murine LN [22].

Urinary MCP-1, VCAM-1 and CXCL16 thus emerge as promising markers of LN, with independent confirmation of these findings by two or more groups. However, few studies have been designed to assess urinary biomarker levels in relation to the status of concurrent renal pathology in LN. To address this lack of data, 74 SLE patients with biopsy-proven LN seen at UT Southwestern Medical Center, Dallas, TX, USA were studied for urinary levels of MCP-1, CXCL16 and VCAM-1. Of these, 24 patients had urine samples drawn at the same time as the renal biopsy was performed. This offered us the unique opportunity to identify the markers that were most predictive of the class of glomerulopathy as well as renal pathology activity/chronicity indices in LN. In addition, we also included as controls patients with anti-neutrophil cytoplasmic antibodies-associated glomerulonephritis (ANCA-GN), focal segmental glomerulosclerosis (FSGS), membranous nephropathy and minimal-change disease, in order to assess the specificity of any observed associations with LN.

## Materials and methods

### Study subjects

The study was approved by the UT Southwestern Institutional Review Board. Patients with LN who were followed up at the outpatient clinic or were hospitalized at UT Southwestern Medical Center were consented and enrolled in the study if they had biopsy-proven LN during the course of their follow-up. Included were subjects who underwent kidney biopsy during the study enrollment period, before they were started on immunosuppressive treatment for their LN.

SLE disease activity at the time of sample procurement was calculated using the SELENA Systemic Lupus Erythematosus Disease Activity Index (SLEDAI) [23]. Renal SLEDAI refers to the total of all renal components used to calculate the SLEDAI. Patients with renal SLEDAI > 0 (that is, patients with hematuria, pyuria, proteinuria or red blood cell casts) were therefore classified as having active renal SLE. Patient characteristics are detailed in Table 1. Although the renal pathology glomerulonephritis class is detailed for all patients in Table 1, only 24 of the biopsies were captured on the day of urine procurement for the biomarker assays. Samples were also obtained from healthy volunteers as a control group, with an average age of 37 (not significantly different from that of the SLE patients), comprising 31% females, 92% Asians and 8% Caucasians. In addition, samples were also obtained from 22 patients with other glomerular diseases: six patients with ANCA-GN (two females, age 52 to 64 years, median protein:creatinine ratio 1.44 mg/mg, serum creatinine 0.95 to 3.82 mg/dl), six patients with FSGS (two females, age 19 to 50 years, median protein:creatinine ratio 3.02 mg/mg, serum creatinine 0.55 to 3.96 mg/dl), three patients with minimal change disease (one female, age 24 to 53 years, protein:creatinine ratio 0.03 to 0.24 mg/mg, serum creatinine 0.48 to 0.94 mg/dl), and seven patients with membranous nephropathy (seven males, age 23 to 60 years, median protein:creatinine ratio 1.08 mg/mg, serum creatinine 0.83 to 2.95 mg/dl). After obtaining written consent, urine samples were collected, aliquoted and frozen at -20°C.

**Table 1:** Demographics and clinical characteristics

	Nonactive disease	Active disease	Total
Patients	14	60	74
Age (years)	37.5 (26.2 to 37.5)	34.0 (28.0 to 40.0)	35.0 (27.5 to 42.0)
Female/male	11/3	52/8	63/11
Race: African American, Hispanic, White	7, 7, 0	26, 27, 7	33, 34, 7
SLEDAI	2 (0 to 5.25)	10 (4 to 17)	10 (4 to 16)
Renal SLEDAI	0	8 (4 to 8)	4 (4 to 8)
**Renal pathology**			
World Health Organization classification			
II	4	8	12
III	1	16	17
IV	3	25	28
V	6	7	13
Unknown	0	3	3
Protein:creatinine ratio	0.190 ± 0.040	1.536 ± 0.229*	1.278 ± 0.195
Serum creatinine	1.422 ± 0.403	1.738 ± 0.191	1.677 ± 0.172
Positive anti-dsDNA (total tested)	4 (11)	30 (50)	34 (61)
Hypocomplementemia (total tested)	9 (11)	35 (46)	44 (57)
Current medications			
Prednisone	10	47	57
Mycophenolic acid	5	24	29
Cyclophosphamide	0	3	3
Azathioprine	2	3	5
Methotrexate	1	1	2
Cyclosporin A	0	1	1
Angiotensin blocking agents	9	32	41
Hydrochloroquine	7	26	33

Data presented as *n*, median (interquartile range) or mean ± standard error. Nonactive disease, renal Systemic Lupus Erythematosus Disease Activity Index (SLEDAI) = 0; active disease, renal SLEDAI > 0. dsDNA, double-stranded DNA. *P < 0.01, active versus nonactive.

### Biomarker assays

ELISA kits for assaying MCP-1, CXCL16 or VCAM-1 were purchased from R&D Laboratories (Minneapolis, Minnesota, USA) or Cayman Chemicals (Ann Arbor, Michigan, USA) and were used as indicated by the manufacturer (human CXCL16 Duo Set, catalog number DY1164; human VCAM1 Duo Set, catalog number DY809; human MCP1 DY 279 and creatinine assay kit, catalog number 500701). All urine samples were diluted 1:1 or more for the ELISA, and the concentrations of the respective molecules were ascertained from standard curves constructed using manufacturer-supplied standards. The urine protein:creatinine ratio was used as an estimate of 24-hour proteinuria, and the urinary levels of all three molecules assayed were normalized against the corresponding urine creatinine levels.

### Renal pathology

Renal pathology was reported using the standardized International Society of Nephrology/Renal Pathological Society classification [24]. In addition, the activity index (AI) and the chronicity index (CI) were calculated in all kidney biopsies exhibiting class III or class IV LN using well-established guidelines [25]. The histological parameters used to calculate composite scores for the AI were cellular crescents, fibrinoid necrosis and karyorrhexis, endocapillary proliferation, leukocytic exudation, hyaline deposits and interstitial infiltration. Each feature, if present, contributed a score of 3, except fibrinoid necrosis and crescents that were weighted twice as much; hence the maximum possible score is 24. The CI was scored based on the presence of glomerular sclerosis, fibrous crescents, interstitial fibrosis and tubular atrophy. The maximum possible score is 12 since each morphological parameter contributes 3 points to the index. Although the renal pathology glomerulonephritis class is detailed for all patients in Table 1, only 24 of the biopsies were captured on the day of urine procurement for the biomarker assays.

### Biostatistical analysis

Groups were compared against each other using the Student's *t *test where the data were normally distributed. Otherwise, the nonparametric Mann-Whitney *U *test was used. Statistical tests including correlation coefficients (*R*) were calculated using Graph Pad Prism. *P *< 0.05 was considered significant.

## Results

There were 74 LN patients, 13 healthy volunteers and 22 disease controls enrolled in this study. One patient was excluded since he had progressed to end-stage renal disease and was on dialysis at the time of sample procurement. There were 12 males and 61 females. Thirty-three participants were African Americans and 33 participants were Hispanics, with the remaining seven being whites. All patient characteristics are detailed in Table 1. The mean age at the time of study participation was 35.7 years. Of the patients enrolled, 24 patients had a concomitant kidney biopsy performed at the time of urine collection. Urine samples were collected before initiating any new immunosuppressant therapy for their LN. Among these 24 patients, four patients were diagnosed with class II LN, five patients with class III LN, 11 patients with class IV LN, and the remaining four patients were classified as having class V LN. The results were analyzed in two phases.

**Figure 1:**
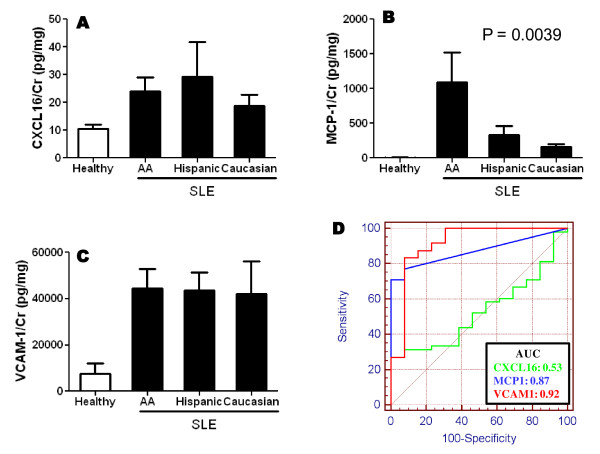
**Elevated levels of urine CXCL16, MCP-1 and VCAM-1 in patients with lupus nephritis**. **(A) **Creatinine (Cr)-normalized urine levels of CXC ligand 16 (CXCL16), **(B) **monocyte chemotactic protein-1 (MCP-1), and **(C) **vascular cell adhesion molecule-1 (VCAM-1) in systemic lupus erythematosus (SLE) patients (*n *= 73) and healthy controls (*n *= 13), segregated according to their ethnicity (AA, African American). **(D) **Receiver operating characteristic curves relating the specificity and sensitivity profiles of the three urinary molecules. AUC, area under curve.

In the first phase we examined the urine biomarker levels in all 73 SLE patients studied, in relation to various clinical parameters, regardless of when the kidney biopsy was carried out. Among the 73 SLE patients, the mean 24-hour urine protein:creatinine ratio was 1.28 ± 0.195 and the mean creatinine-normalized VCAM-1 level was 43,360 ± 5,518 pg/mg. Both markers correlated well with each other (*R *= 0.82, *P *< 0.0001). Although both urinary MCP-1 (mean 542.9 ± 183.5 pg/mg) and CXCL16 (mean 24.8 ± 6.19 pg/mg) were significantly correlated with proteinuria, the correlation was smaller compared with that for urinary VCAM-1 (*r *= 0.73, *P *< 0.0001 and *r *= 0.24, *P *= 0.02 for MCP-1 and CXCL16, respectively). Although all three molecules were elevated in the urine of SLE patients, they showed interesting differences particularly when the patients were segregated based on ethnicity (Figure 1). Whereas CXCL16 and VCAM-1 were elevated in all ethnic groups examined, MCP-1 was elevated most prominently among the African American subjects (Figure 1A, B, C). Overall, urine VCAM-1 (area under curve = 0.92) and MCP-1 (area under curve = 0.87) were better in distinguishing the SLE samples from the healthy control samples (Figure 1D).

**Figure 2:**
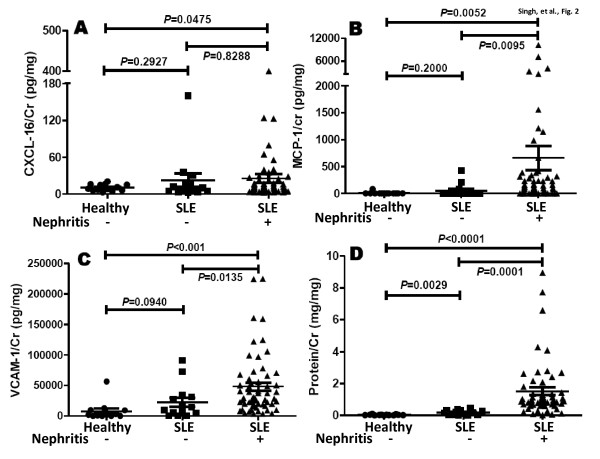
**Elevated levels of urine CXCL16, MCP-1 and VCAM-1 in patients with active disease**. **(A) **Creatinine (Cr)-normalized urine levels of CXC ligand 16 (CXCL16), **(B) **monocyte chemotactic protein-1 (MCP-1), **(C) **vascular cell adhesion molecule-1 (VCAM-1) and **(D) **protein in systemic lupus erythematosus (SLE) patients with inactive disease (*n *= 14), active renal disease (*n *= 59) and healthy controls (*n *= 13). Each dot represents one individual.

**Figure 3:**
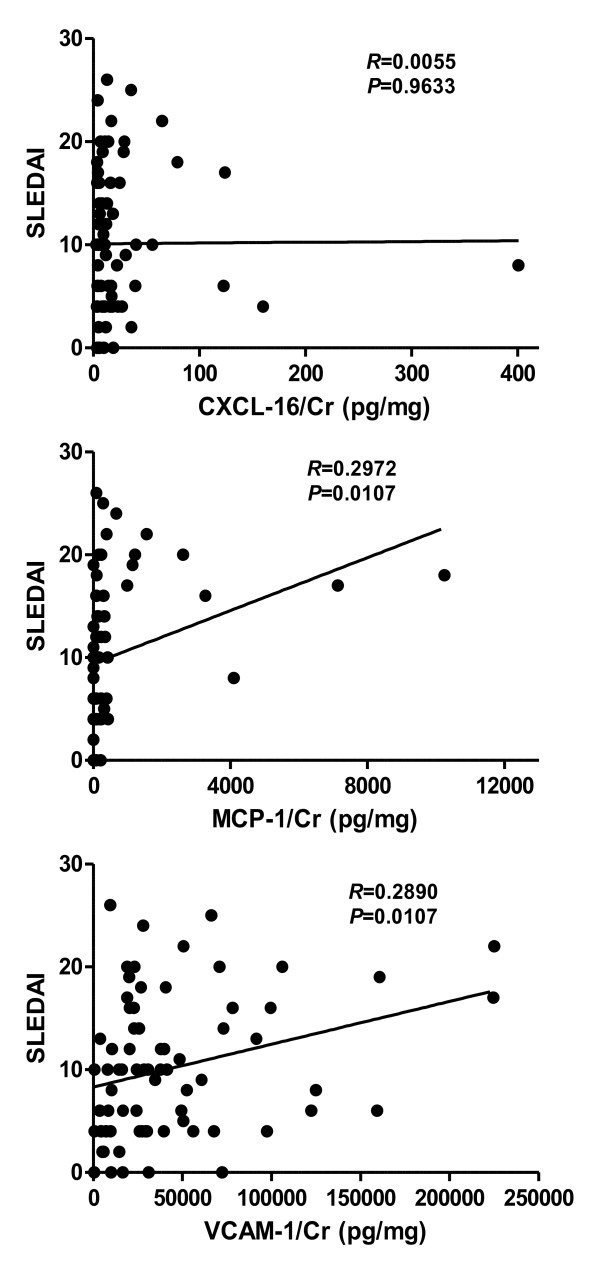
**Correlation plots of urine CXCL16, MCP-1 and VCAM-1 with systemic lupus erythematosus disease activity**. Creatinine (Cr)-normalized urine levels of CXC ligand 16 (CXCL16), monocyte chemotactic protein-1 (MCP-1) and vascular cell adhesion molecule-1 (VCAM-1) in systemic lupus erythematosus patients (*n *= 73) in relation to their disease activity, as scored by their (global) Systemic Lupus Erythematosus Disease Activity Index (SLEDAI). *R*, correlation coefficient.

The levels of these four markers were then examined after segregating the patients based on whether or not they had active renal disease (Table 1). Importantly, patients with active renal disease exhibited statistically significantly higher mean levels of creatinine-normalized MCP-1 and VCAM-1, compared with the other SLE patients (Figure 2B, C); in contrast, urine CXCL16 was not as discriminatory (Figure 2A). The urine protein:creatinine ratios also demonstrated a similar trend, with mean values in active renal SLE, nonactive renal SLE and healthy volunteers being 1.66 ± 0.26, 0.57 ± 0.15 and 0.0045 ± 0.008, respectively (Figure 2D). Similarly, when the urine levels of the three markers were analyzed in relation to the SLEDAI disease activity score, creatinine-normalized MCP-1 and VCAM-1 exhibited statistically significant correlations with the SLEDAI, unlike urine CXCL16 (Figure 3).

To evaluate whether concurrent medications might impact the level of these urinary markers, we first examined whether the medications used were different between the patients with active nephritis and the patients without. As shown in Table 1, the proportions of patients on immunosuppressants, angiotensin-blocking agents or hydrochloroquine were not significantly different between these two groups. We then subdivided each group (active nephritis or nonactive nephritis) into two subgroups - those who received each of the medications listed in Table 1, and those who were not on that specific medication - and compared the levels of these makers between the subgroups, within active patients or nonactive patients, respectively. The only statistically significant difference seen was in the active nephritis group: mean MCP-1 levels in three patients who received cyclophosphomide were lower than those who did not receive it (*P *= 0.03). Among the nonactive nephritis patients, therefore, VCAM-1, CXCL16 and MCP-1 levels and the urine protein:creatinine ratio were not different between the patients who received a specific medication and those who did not. Likewise, among active nephritis patients, VCAM-1 and CXCL16 levels as well as the urine protein:creatinine ratio were not different between the patients who received a specific medication and those who did not.

**Figure 4:**
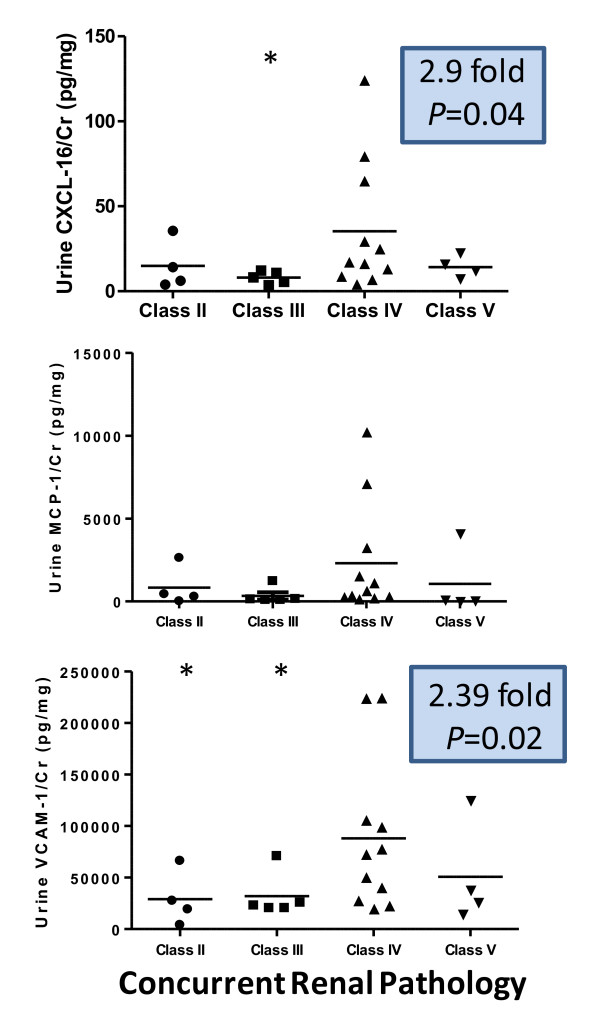
**Elevated levels of urine CXCL16, MCP-1 and VCAM-1 in patients with class IV lupus nephritis**. Creatinine (Cr)-normalized urine levels of CXC ligand 16 (CXCL16), monocyte chemotactic protein-1 (MCP-1) and vascular cell adhesion molecule-1 (VCAM-1) in systemic lupus erythematosus patients (*n *= 24), segregated according to the glomerulonephritis (GN) class that was present in a concurrent renal biopsy specimen obtained at the time of urine procurement. The indicated fold-change and *P *value refer to class IV GN compared with all other GN classes.

**Figure 5:**
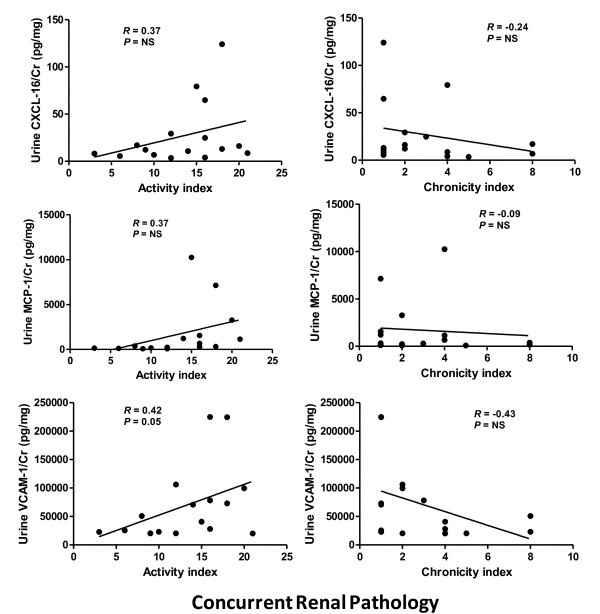
**Elevated urine CXCL16, MCP-1 and VCAM-1 and correlation to renal pathology activity and chronicity indices**. Creatinine (Cr)-normalized urine levels of CXC ligand 16 (CXCL16), monocyte chemotactic protein-1 (MCP-1) and vascular cell adhesion molecule-1 (VCAM-1) in systemic lupus erythematosus patients (*n *= 24), correlated according to the activity index (left) and chronicity index (right) that was scored in a concurrent renal biopsy specimen obtained at the time of urine procurement. NS, not significant; *R*, correlation coefficient.

A second phase of analysis was carried out, focusing on a unique subset of the recruited LN patients. Since the urine samples for 24 of the participating subjects were obtained at the time of kidney biopsy, we correlated the renal pathology with the corresponding levels of the assayed markers. When the urinary levels of the three molecules in patients with class IV LN were compared against the corresponding levels in patients with other classes of LN, all three markers exhibited higher levels in class IV LN and tended to differentiate this class from other classes of LN (Figure 4), with the differences for CXCL16 and VCAM-1 attaining statistical significance. Since the renal pathology specimens from the patients who had class III/IV LN were also scored for the AI and the CI, this allowed us to examine whether the urine levels of the markers were particularly associated with activity changes or chronicity changes. The mean AI and CI scores for the class III + IV LN patients were 13.375 and 2.79, respectively. The urinary expression levels of the three study molecules correlated positively with the AI, although statistical significance was seen only for urine creatinine-normalized VCAM-1 (Figure 5). Similarly, when correlated against the CI, all three markers correlated negatively with this pathological index (Figure 5). VCAM-1 thus emerges as the urinary molecule that is best associated with class IV LN as well as concurrent renal pathology activity in SLE patients.

**Figure 6:**
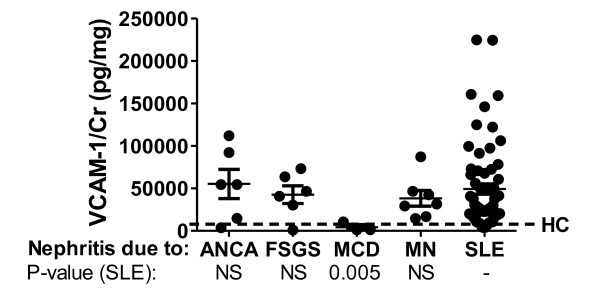
**Urine vascular cell adhesion molecule-1 levels in other nephropathies**. Urine vascular cell adhesion molecule-1 (VCAM-1) levels were assayed and creatinine (Cr)-normalized using urine samples from patients with anti-neutrophil cytoplasmic antibodies-associated (ANCA) glomerulonephritis, patients with focal segmental glomerulosclerosis (FSGS), patients with minimal-change disease (MCD) and patients with membranous nephropathy (MN), as plotted. The basal levels in the healthy controls are represented by the dotted line (HC). The data for the healthy controls and the lupus nephritis patients are drawn from the data shown in Figure 2C. The VCAM-1 levels noted in the different groups are statistically compared with the lupus nephritis group, as indicated below the *x *axis label. NS, not significant; SLE, systemic lupus erythematosus.

Finally, we wanted to assess the disease specificity of urinary VCAM-1. Although our data clearly indicate that patients with active LN had the highest levels of urinary VCAM-1, whether other types of inflammatory or non-inflammatory nephropathies may also be associated with elevated VCAM-1 levels is unclear. Interestingly, the urinary levels of VCAM-1 in patients with ANCA-GN, FSGS and membranous nephropathy were not statistically different from the urine levels of VCAM-1 in patients with active LN (Figure 6). In contrast, patients with minimal-change disease had low levels of VCAM-1, comparable with those found in the healthy controls.

## Discussion

The management of LN remains an intricate problem [26,27] since the markers currently used to prompt a kidney biopsy or to predict treatment response or nephritic flare-ups are inaccurate and tend to delay the diagnosis and further interventions [5,28]. Moreover, patients with silent LN may not exhibit abnormalities of traditionally used markers such as urinary sediment, proteinuria or serum creatinine [29-31]. Similarly, the elevation of markers such as proteinuria may not always reflect disease activity in the kidney [32]. Furthermore, currently used markers may not accurately differentiate between active LN and damaged kidney from past inflammation. There is therefore an urgent need for better markers that can overcome the shortcomings associated with the monitoring of serum creatinine, the urine protein:creatinine ratio and urinary sediment. In the present study, we examined the disease relevance of three previously reported emerging biomarkers - urinary VCAM-1, MCP-1 and CXCL16 - in relation to the more traditionally used clinical parameters and renal pathology indices. The study population is unique since all patients had kidney biopsy performed at some stage during follow-up, with 24 of the patients having a renal biopsy at the time of sample collection. When compared against 24-hour estimated proteinuria, all three molecules exhibited significant correlation but VCAM-1 and MCP-1 showed the strongest correlation. Importantly, urinary VCAM-1 and MCP-1 levels were significantly elevated in the active renal disease group as compared with healthy controls and patients with inactive renal disease. The same pattern was observed when the markers were correlated against SLEDAI disease activity scores - both urinary MCP-1 and VCAM-1 demonstrated strong correlation with disease activity.

When the urinary levels of the three molecules were analyzed in patients who had a concurrent kidney biopsy performed, the levels of all three molecules were elevated in class IV LN and tended to differentiate class IV patients from the subjects with other classes of LN, again with VCAM-1 being the most discriminatory. This is an important finding since class IV LN has ominous prognosis, warranting prompt and aggressive treatment. An interesting finding emerged when we examined the relationship of these markers against the corresponding renal pathology AI in concurrently performed renal biopsies. All three molecules correlated positively with the AI, but only the correlation for urine VCAM-1 was statistically significant. Since the AI signifies severe but potentially reversible inflammation, this is an important finding with respect to disease monitoring and treatment. Similarly, all three markers showed a trend towards a negative correlation with CI - high chronicity indices denote late-stage fibrosis, minimal inflammation and irreversible damage to the kidney, all of which bode poorly even with treatment.

Taken together, these findings suggest that urinary VCAM-1 may be a reliable indicator of the activity:chronicity ratios that mark the underlying renal pathology in LN. In comparison with VCAM-1, urine MCP-1 performed modestly in its predictive capacity while CXCL16 was even less promising as a disease marker. VCAM-1 is an important adhesion molecule involved in the key process of leukocytic migration into the kidneys. Leukocytic infiltration is a hallmark of severe renal disease and one of the morphological features contributing to the elevated AI in LN. Since LN is involved in the acute phase of inflammation when leukocytic infiltration is ongoing and since VCAM-1 levels are likely to recede with reduced activity and when chronicity sets in, tracking VCAM-1 levels longitudinally may help monitor disease activity over time.

However, our findings indicate that elevated urinary VCAM-1 is not specific for SLE; rather, it appears to be a marker of renal injury, since the levels are also elevated in other types of inflammatory nephritis (for example, ANCA-GN) as well as nephropathies not typically associated with inflammation (for example, FSGS). Although the numbers of each type of renal disease control are rather limited, and are not completely matched with the LN patients with respect to renal function, age or gender, these preliminary findings warrant a more thorough investigation of urinary VCAM-1 levels in other renal diatheses. In addition, the clinical utility of using urine VCAM-1 to track disease progression in ANCA-GN, FSGS and membranous nephropathy should also be carefully examined in future studies. To this end, the increased expression of VCAM-1 either within the kidneys or in the urine has already been documented previously in patients with ANCA-GN, FSGS and membranous nephropathy, in resonance with our findings [33-36]. Interestingly, the level of VCAM-1 expression appears to correlate with the degree of disease in ANCA-GN and FSGS [33,36].

The clinical use of urinary VCAM-1 clearly must be coupled with a second marker that can accurately predict chronicity changes within the kidney. In this light, although markers such as transforming growth factor beta offer promise [37], several other candidate biomarkers are currently being evaluated in several laboratories. These emerging candidates hold great promise, given that none of the currently employed yardsticks is able to accurately predict the degree of renal pathology activity and/or chronicity in LN. What is needed next is a longitudinal study in which LN patients are monitored serially for urine levels of VCAM-1, as well as a marker of renal pathology chronicity, coupled with traditional disease markers, in order to establish whether non-invasive markers can be used successfully to accurately predict underlying renal disease and the clinical course in LN.

## Conclusion

Three urinary molecules and concurrent renal pathology were examined in SLE. Urinary VCAM-1 emerges as a good indicator of the renal pathology AI in patients with LN.

## Abbreviations

AI: activity index; ANCA-GN: anti-neutrophil cytoplasmic antibodies-associated glomerulonephritis; CI: chronicity index; CXCL16: CXC ligand 16; ELISA: enzyme-linked immunosorbent assay; FSGS: focal segmental glomerulosclerosis; LN: lupus nephritis; MCP-1: monocyte chemotactic protein-1; SLE: systemic lupus erythematosus; SLEDAI: Systemic Lupus Erythematosus Disease Activity Index; VCAM-1: vascular cell adhesion molecule-1.

## Competing interests

This work was supported in part by the George M. O'Brien Kidney Research Core Center (NIH P30DK079328, NIH RO1 AR050812, R01 DK 081872) and by the Lupus Research Institute. The authors declare that they have no competing interests.

## Authors' contributions

SS, TW, CX, KV and JH executed the studies. SS, TW, TM, HBO and CA contributed to data analysis and writing the manuscript. XJZ performed the pathological analysis. CP and RS contributed patient material and data. CP, RS and CM contributed to planning the study and preparing the manuscript. All authors read and approved the final manuscript.

## References

[B1] Appel GRJ D'Agati V The Kidney 2007 8 Philadelphia, PA: Saunders Elsevier

[B2] US Renal Data System Incidence of reported ESRD, by primary diagnosis 2002-2006 Combined 2008

[B3] Fiehn C Hajjar Y Mueller K Waldherr R Ho AD Andrassy K Improved clinical outcome of lupus nephritis during the past decade: importance of early diagnosis and treatment Ann Rheum Dis 2003 62 435 439 10.1136/ard.62.5.435 12695156 PMC1754523

[B4] Faurschou M Starklint H Halberg P Jacobsen S Prognostic factors in lupus nephritis: diagnostic and therapeutic delay increases the risk of terminal renal failure J Rheumatol 2006 33 1563 1569 16881113

[B5] Esdaile JM Joseph L MacKenzie T Kashgarian M Hayslett JP The benefit of early treatment with immunosuppressive agents in lupus nephritis J Rheumatol 1994 21 2046 2051 7869308

[B6] Rovin BH Song H Birmingham DJ Hebert LA Yu CY Nagaraja HN Urine chemokines as biomarkers of human systemic lupus erythematosus activity J Am Soc Nephrol 2005 16 467 473 10.1681/ASN.2004080658 15601744

[B7] Li Y Tucci M Narain S Barnes EV Sobel ES Segal MS Richards HB Urinary biomarkers in lupus nephritis Autoimmun Rev 2006 5 383 388 10.1016/j.autrev.2005.10.006 16890891

[B8] Enghard P Riemekasten G Immunology and the diagnosis of lupus nephritis Lupus 2009 18 287 290 10.1177/0961203308099632 19276295

[B9] Wu T Xie C Wang HW Zhou XJ Schwartz N Calixto S Mackay M Aranow C Putterman C Mohan C Elevated urinary VCAM-1, P-selectin, soluble TNF receptor-1, and CXC chemokine ligand 16 in multiple murine lupus strains and human lupus nephritis J Immunol 2007 179 7166 7175 17982109 10.4049/jimmunol.179.10.7166

[B10] Ikeda Y Fujimoto T Ameno M Shiiki H Dohi K Relationship between lupus nephritis activity and the serum level of soluble VCAM-1 Lupus 1998 7 347 354 10.1191/096120398678920172 9696139

[B11] Ilic T Mitic I Durdevic-Mirkovic T Vuckovic B Milic B Popovic M Correlation between sera levels of sICAM-1 and sVCAM-1 and severity of kidney lesions in patients with lupus nephritis Med Pregl 2007 60 Suppl 2 128 132 18928178

[B12] Wada T Yokoyama H Su SB Mukaida N Iwano M Dohi K Takahashi Y Sasaki T Furuichi K Segawa C Hisada Y Ohta S Takasawa K Kobayashi K Matsushima K Monitoring urinary levels of monocyte chemotactic and activating factor reflects disease activity of lupus nephritis Kidney Int 1996 49 761 767 10.1038/ki.1996.105 8648917

[B13] Molad Y Miroshnik E Sulkes J Pitlik S Weinberger A Monselise Y Urinary soluble VCAM-1 in systemic lupus erythematosus: a clinical marker for monitoring disease activity and damage Clin Exp Rheumatol 2002 20 403 406 12102480

[B14] Noris M Bernasconi S Casiraghi F Sozzani S Gotti E Remuzzi G Mantovani A Monocyte chemoattractant protein-1 is excreted in excessive amounts in the urine of patients with lupus nephritis Lab Invest 1995 73 804 809 8558841

[B15] Rovin BH Birmingham DJ Nagaraja HN Yu CY Hebert LA Biomarker discovery in human SLE nephritis Bull NYU Hosp Jt Dis 2007 65 187 193 17922668

[B16] Tesch GH Maifert S Schwarting A Rollins BJ Kelley VR Monocyte chemoattractant protein 1-dependent leukocytic infiltrates are responsible for autoimmune disease in MRL-Fas(lpr) mice J Exp Med 1999 190 1813 1824 10.1084/jem.190.12.1813 10601356 PMC2195716

[B17] Marks SD Williams SJ Tullus K Sebire NJ Glomerular expression of monocyte chemoattractant protein-1 is predictive of poor renal prognosis in pediatric lupus nephritis Nephrol Dial Transplant 2008 23 3521 3526 10.1093/ndt/gfn270 18495743

[B18] Wuthrich RP Vascular cell adhesion molecule-1 (VCAM-1) expression in murine lupus nephritis Kidney Int 1992 42 903 914 10.1038/ki.1992.367 1280699

[B19] Zhu J Liu X Xie C Yan M Yu Y Sobel ES Wakeland EK Mohan C T cell hyperactivity in lupus as a consequence of hyperstimulatory antigen-presenting cells J Clin Invest 2005 115 1869 1878 10.1172/JCI23049 15951839 PMC1143586

[B20] Pizarro S Monarrez Espino J Ruiz A Jara LJ Nava A Riebeling-Navarro C Soluble vascular cell adhesion molecule-1 indicates SLE disease activity and specific organ involvement Rev Alerg Mex 2007 54 189 195 18693542

[B21] Spronk PE Bootsma H Huitema MG Limburg PC Kallenberg CG Levels of soluble VCAM-1, soluble ICAM-1, and soluble E-selectin during disease exacerbations in patients with systemic lupus erythematosus (SLE); a long term prospective study Clin Exp Immunol 1994 97 439 444 7521807 10.1111/j.1365-2249.1994.tb06107.x PMC1534867

[B22] Teramoto K Negoro N Kitamoto K Iwai T Iwao H Okamura M Miura K Microarray analysis of glomerular gene expression in murine lupus nephritis J Pharmacol Sci 2008 106 56 67 10.1254/jphs.FP0071337 18187931

[B23] Petri M Magder L Classification criteria for systemic lupus erythematosus: a review Lupus 2004 13 829 837 10.1191/0961203304lu2019oa 15580978

[B24] Weening JJ D'Agati VD Schwartz MM Seshan SV Alpers CE Appel GB Balow JE Bruijn JA Cook T Ferrario F Fogo AB Ginzler EM Hebert L Hill G Hill P Jennette JC Kong NC Lesavre P Lockshin M Looi LM Makino H Moura LA Nagata M The classification of glomerulonephritis in systemic lupus erythematosus revisited J Am Soc Nephrol 2004 15 241 250 10.1097/01.ASN.0000108969.21691.5D 14747370

[B25] Austin HA Muenz LR Joyce KM Antonovych TT Balow JE Diffuse proliferative lupus nephritis: identification of specific pathologic features affecting renal outcome Kidney Int 1984 25 689 695 10.1038/ki.1984.75 6482173

[B26] Singh S Saxena R Lupus nephritis Am J Med Sci 2009 337 451 460 10.1097/MAJ.0b013e3181907b3d 19390431

[B27] Houssiau FA Management of lupus nephritis: an update J Am Soc Nephrol 2004 15 2694 2704 10.1097/01.ASN.0000140218.77174.0A 15466274

[B28] Cameron JS Lupus nephritis J Am Soc Nephrol 1999 10 413 424 10215343 10.1681/ASN.V102413

[B29] Font J Torras A Cervera R Darnell A Revert L Ingelmo M Silent renal disease in systemic lupus erythematosus Clin Nephrol 1987 27 283 288 3497000

[B30] Zabaleta-Lanz M Vargas-Arenas RE Tapanes F Daboin I Atahualpa Pinto J Bianco NE Silent nephritis in systemic lupus erythematosus Lupus 2003 12 26 30 10.1191/0961203303lu259oa 12587823

[B31] Wada Y Ito S Ueno M Nakano M Arakawa M Gejyo F Renal outcome and predictors of clinical renal involvement in patients with silent lupus nephritis Nephron Clin Pract 2004 98 c105 c111 10.1159/000081551 15627787

[B32] Bajaj S Albert L Gladman DD Urowitz MB Hallett DC Ritchie S Serial renal biopsy in systemic lupus erythematosus J Rheumatol 2000 27 2822 2826 11128670

[B33] Arrizabalaga P Sole M Iglesias C Escaramis G Ascaso C Renal expression of ICAM-1 and VCAM-1 in ANCA-associated glomerulonephritis - are there differences among serologic subgroups? Clin Nephrol 2006 65 79 86 16509455 10.5414/cnp65079

[B34] Abd-Elkareem MI Al Tamimy HM Khamis OA Abdellatif SS Hussein MR Increased urinary levels of the leukocyte adhesion molecules ICAM-1 and VCAM-1 in human lupus nephritis with advanced renal histological changes: preliminary findings Clin Exp Nephrol 2010 14 548 557 10.1007/s10157-010-0322-z 20714774

[B35] Zhang Q Zeng C Fu Y Cheng Z Zhang J Liu Z Biomarkers of endothelial dysfunction in patients with primary focal segmental glomerulosclerosis Nephrology (Carlton) 2012 17 338 345 10.1111/j.1440-1797.2012.01575.x 22295953

[B36] Honkanen E von Willebrand E Teppo AM Tornroth T Gronhagen-Riska C Adhesion molecules and urinary tumor necrosis factor-alpha in idiopathic membranous glomerulonephritis Kidney Int 1998 53 909 917 9551397 10.1111/j.1523-1755.1998.00833.x

[B37] Saxena V Lienesch DW Zhou M Bommireddy R Azhar M Doetschman T Singh RR Dual roles of immunoregulatory cytokine TGF-β in the pathogenesis of autoimmunity-mediated organ damage J Immunol 2008 180 1903 1912 18209088 10.4049/jimmunol.180.3.1903 PMC2291535

